# Rab GTPases as Modulators of Vascular Function

**DOI:** 10.3390/cells11193061

**Published:** 2022-09-29

**Authors:** Somasundaram Raghavan, Masuma Akter Brishti, M. Dennis Leo

**Affiliations:** Department of Pharmaceutical Sciences, University of Tennessee Health Science Center, Memphis, TN 38163, USA

**Keywords:** Rab GTPases, endothelial cells, vascular smooth muscle

## Abstract

Rab GTPases, the largest family of small GTPases, are ubiquitously expressed proteins that control various aspects of cellular function, from cell survival to exocytosis. Rabs cycle between the GDP-bound inactive form and the GTP-bound active form. When activated, specific Rab GTPase-positive vesicles mediate cellular networks involved in intracellular trafficking, recycling, and/or exocytosis of cargo proteins. Dysfunctional Rab signaling pathways have been implicated in various disease processes. The precise cellular functions of several members of the Rab GTPase family are still unknown. A lack of pharmacological tools and the lethality of gene knockouts have made more detailed characterizations of their protein interaction networks difficult. Nevertheless, available evidence suggests that these proteins are vital for normal cell function. Endothelial and smooth muscle cells control vascular lumen diameter and modulate blood flow. Endothelial cells also secrete several pro- and antithrombotic factors and vasoactive substances to coordinate local inflammatory responses and angiogenesis. Rab GTPase function in endothelial cells has been relatively well-explored, while only a handful of reports are available on these proteins in vascular smooth muscle. This review summarizes the present knowledge on Rab GTPases in the vasculature.

## 1. Introduction

The organization of eukaryotic cells enables complex biochemical reactions to proceed with unparalleled precision. While all synthesized proteins pass through the same cellular machinery, their distribution within the cell is determined by the specific roles they play. Accordingly, proteins are targeted to organelles such as the mitochondria or lysosomes, to the plasma membrane, or are packaged within vesicles for exocytosis. This ‘targeted’ trafficking of proteins to different cellular compartments and organelles is primarily controlled by a group of proteins called Rab GTPases [1,2,3]. With nearly 70 family members, Rab GTPases are the largest group in the Ras family of small GTPases [1]. Rabs cycle between two conformations, the inactive guanosine diphosphate (GDP)-bound form and the active guanosine triphosphate (GTP)-bound form. Rab ‘activation’ occurs when guanine nucleotide exchange factors (GEFs) convert the GDP-bound form of Rabs to the GTP-bound form [1,2,3]. Once activated, Rab GTPases then coordinate a series of highly orchestrated events to shuttle their cargo proteins to their intended destinations [4]. Given their vital role in cell function, Rabs are extensively regulated by different pathways, including mRNA compartmentalization, prenylation, phosphorylation, and ubiquitination [1,5]. While the functions of several Rabs have been elucidated in detail, many more are still unknown. A significant disadvantage for researchers studying Rab GTPases is the lack of selective pharmacological tools to modulate protein function, in addition to only a few known viable mouse knockouts [1,2,3,4,6]. Rab GTPases are involved in diverse cellular events, from cell cycle regulation to synapse and secretory function [1,2,3,4]. Although Rabs first emerged as obligatory players in the development of several cancers, they have since been implicated in various diseases ranging from cardiovascular disease and diabetes to Parkinson’s and several other neurological disorders [1,2,3,4,7]. While extensive reviews on the workings of Rab GTPases are available elsewhere [1,2,3,4,6,7], we focus on the current knowledge of Rab GTPase function in the vasculature, specifically in endothelial and smooth muscle cells.

Vascular endothelial cells, which line the inner walls of arteries, capillaries, and veins, are polarized cells with a luminal side exposed to blood and a basolateral side in contact with surrounding cells, including smooth muscle cells [8,9,10]. Endothelial cells play a vital role in modulating vascular lumen diameter through the release of vasoactive substances, secrete several coagulant and antithrombotic factors, and coordinate local inflammatory response and angiogenesis [8,9,10]. Vascular smooth muscle cells simultaneously respond to blood-borne or endothelial-cell-released signaling mediators to constrict or dilate blood vessels, thereby modulating blood flow through different regions of the body [11,12,13]. The functions of both these cells are controlled by signaling pathways originating from a wide range of receptors and often cell-specific proteins. Accumulating evidence has drawn attention to Rab GTPases as the coordinators of these intricate signaling pathways. In this minireview, we explore the function of Rab GTPases involved in vascular homeostasis.

## 2. Rab GTPase Signaling in Endothelial Cells

### 2.1. Rab GTPases Support the Secretory Function of Endothelial Cells

Endothelial cell secretion of the procoagulant protein von Willebrand factor (vWF) follows a complex series of protein packaging and trafficking steps that involves several Rab proteins and their association with rod-shaped endothelial-specific storage organelles called Weibel–Palade bodies (WPBs). The Rabs involved in WPB exocytosis, termed ‘secretory Rabs’, include Rab3 isoforms (3B and 3D) and Rab27A and appear to be essential for this endothelial-cell-specific function. One of these, Rab27A, was recruited only to mature WPBs in endothelial cells, a phenomenon that was shown to occur even in an exogenous expression system [14]. Here, the wild-type vWF expressed in HEK-293 cells could still recruit the secretory Rab27A to their vicinity [14]. Similar to Rab27A, another protein called ‘Myosin VIIA and Rab Interacting Protein’ (MyRIP) was found to be localized only to mature WPBs, suggesting that it had a role in vWF exocytosis [15]. In human umbilical vein endothelial cells (HUVECs), MyRIP was found to be the functional effector of Rab27A, and together they served to anchor WPBs to actin strands near the cell periphery to serve as a secretory ‘checkpoint’, which was suggested to prevent the immature vWF from being exocytosed [15]. A second Rab27A effector, Slp4-a (granuphilin), was later discovered to be a positive regulator of WPB exocytosis, and the interplay between MyRIP and Slp4-a appeared to control the intensity of vWF release [16]. A more detailed full-screen Rab GTPase analysis performed in HUVECs revealed that at least five different Rabs localize with WPBs, which include Rab27, Rab3, Rab15, Rab33A, and Rab37 [17]. Of these, single siRNA-mediated knockdown of either Rab27A, Rab3A, Rab3D, or Rab15 reduced vWF secretion by only ~50%, whereas Rab33 and Rab37 knockdown did not have any effect on vWF secretion [17]. Together, the data suggested that Rab27, Rab3, and Rab15 were the critical Rabs required for vWF secretion and mostly functioned cooperatively with each other [17]. The role of Rab33 and 37 in the vWF secretory process has not yet been determined. In another study, Rab3D was found to be associated only with vWF-positive WPBs but not with antithrombogenic, tissue-type plasminogen activator (tPA)-containing granules [18]. This suggested that at least Rab3D function was specifically related to vWF secretion, and it was proposed that there was possible compartmentalization between prothrombotic and antithrombotic factor secretory vesicles in endothelial cells [18]. Recently, the MAP-kinase activating death domain (MADD), also called DENN (Differentially Expressed in Normal and Neoplastic cells), which was considered a Rab3 guanine exchange protein (Rab3GEP), was identified as a master regulator of WPB function in endothelial cells [19]. MADD controlled the recruitment of Rab27A, 3B, and 3D to WPBs and thereby regulated the exocytosis of the vWF [19]. Contrary to these Rabs, Rab35 appears to be a stand-alone and novel regulator of WPB exocytosis [20]. By overexpressing several Rab GTPase-activating proteins (RabGAPs) in HUVECs and measuring vWF release, investigators uncovered that a RabGAP called TBC1 Domain Family Member 10A (TBC1D10A) interacted with Rab35 to inhibit histamine-evoked, Ca^2+^-dependent WPB exocytosis [20]. 

**Figure 1:**
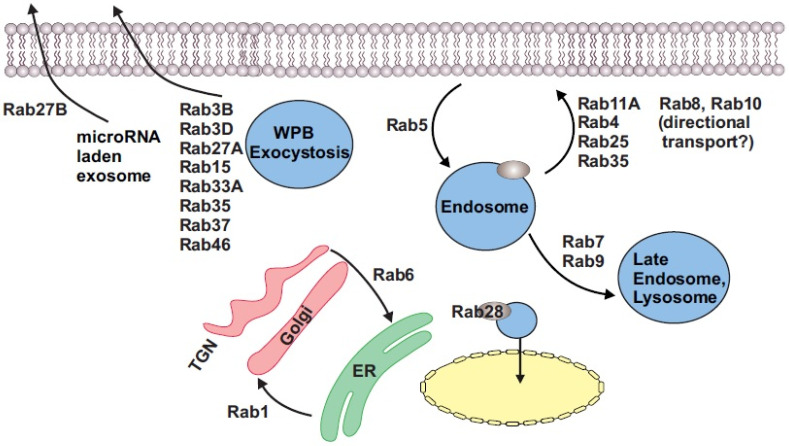
Rab GTPases and their known functions in endothelial and smooth muscle cells.

Endothelial cells also appear to possess secretory mechanisms for specific molecules such as microRNA (miRs). For example, in HUVECS, it was uncovered that a distinct secretory pathway mediated by both Rab7A and 27B was involved in the secretion of miR-143-laden exosomes [21]. miR-143 then likely affected neighboring smooth muscle phenotypic switching [21]. Overall, Rab GTPases are involved in nearly every facet of the endothelial cell exocytosis process (Figure 1).

### 2.2. Rab GTPases Are Involved in Endothelial Cell Peripheral/Surface Trafficking, Permeability, and Proliferation

Following their exit from the trans-Golgi network, most proteins destined to the plasma membrane traverse one or more Rab-positive vesicles over the course of their journey to arrive at their final cellular destinations. For instance, Rab1 promoted β2-adrenergic receptor (β2AR) cell surface expression in pulmonary microvascular endothelial cells [22]. Rab1WT overexpression significantly attenuated lipopolysaccharide (LPS, *E. coli*, 0111:B4)-induced hyperpermeability of pulmonary endothelial cells by increased β2AR trafficking to the cell surface [22]. Since β2AR stimulation activates cAMP-dependent protein kinase A (PKA) signaling, which in turn is critical for vWF secretion [23], Rab1 could have an indirect role to play in endothelial cell secretory pathways. *Fuyuan Xingnao*, a traditional Chinese herbal formulation, activated the Rab1/AT1R (angiotensin II type 1 receptor) signaling pathway to increase AT1R surface expression and trigger brain microvascular endothelial cell proliferation [24]. Similar to AT1R, a few plasma membrane-localized endothelial ion channels have also been reported to be trafficked by Rabs. Rab5 endocytosed K_Ca_2.3 (KCNN3, small-conductance Ca^2+^-activated K^+^ channel-3) from the cell surface, which then interacted with Rab35 and was quickly recycled back to the cell surface within ~5 min [25,26]. In contrast, K_Ca_3.1 (KCNN4) was degraded after being internalized and not recycled [25,26]. Thus, Rab GTPases are also known to regulate endothelial surface receptor density; however, knowledge is scarce on this topic.

Incidentally, the secretory Rabs, Rab3A, 3B, and Rab27A, along with the trafficking Rabs, Rab8A, and Rab11A, were also found to be involved in endothelial tubulogenesis in 3D collagen matrices with siRNA-induced suppression of these proteins inhibiting the process [27]. In contrast, these investigators showed that the knockdown of Rab5A had no effect, while the knockdown of Rab3D stimulated lumen formation [27]. Here, combined knockdown of Rab8A and Rab11A, Rab8A and Rab27A, and Rab27A, and Caveolin1 had a more significant effect on blocking tubulogenesis than individual suppression of these proteins [27]. Another essential component in angiogenesis is vascular endothelial growth factor receptor-2 (VEGFR2, Kdr). VEGF and the signaling initiated through its receptor VEGFR2 are critical in controlling sprouting angiogenesis of endothelial cells [28,29,30]. To modulate VEGFR2 signaling, the receptor is cycled through various endosomal compartments. Several Rabs and Rab effector proteins have been implicated in this, including Rab4, Rab5, Rab7, and Rab11. While Rab5 controls receptor endocytosis, Rab4 and Rab11 have been shown to recycle the receptor back to the plasma membrane for further signaling, while Rab7 shuttles the receptor to the lysosome for degradation [31,32,33]. Recycling of the VEGFR2 receptor through a Rab4A/Rab11A-mediated pathway is vital in preventing ‘receptor shedding’ where the N-terminal extracellular part of the protein is cleaved and released [34]. Interaction of Rab4 with a protein called RabEP2 helps maintain VEGFR2 expression at the cell surface [35]. The absence of RabEP2 diverts the Rab4 vesicles to a Rab7-directed lysosomal pathway [35]. Rab7 was upregulated in human atherosclerotic plaques and aortic endothelial cells of rabbits fed a high-fat diet, suggesting a role in atherogenesis [36]. In addition, VEGFR1(Flt1) signaling in endothelial cells was shown to regulate Rab4-mediated integrin recycling and contribute to angiogenesis [37]. Similar to VEGFR2, CD93, a single-pass membrane protein, is upregulated in hyperproliferative endothelial cells, especially in cancer [38]. CD93 forms a protein complex with the matrix protein multimerin-2 and β1 integrin within Rab5C-positive endosomal compartments to recycle it back to the cell surface [39]. Rab6, Rab8, and Rab10 are theorized to direct vesicular trafficking to the apical side of the endothelial cell during lumen development [40] (Figure 1). In HUVECS, Rab13 blocked mTOR signaling by interacting with growth factor receptor-bound protein 2 (Grb2) to induce autophagy [41]. These investigations provide evidence that endothelial Rabs are actively involved in endothelial cell proliferation, angiogenesis, and autophagy. 

Endothelial cells also maintain the endothelial barrier function to regulate vascular permeability. VE-cadherin is a protein that forms the adherens junctions in endothelial cells, which helps maintain a limiting endothelial barrier. To increase nutrient flow, aid in the inflammatory response, or regulate angiogenesis, there is a concerted loosening of the endothelial barrier by modulation of the expression of proteins such as VE-cadherin that are part of its function. The novel endosome adaptor protein, p18, is essential for VE-cadherin recycling to improve pulmonary endothelial barrier function [42]. During neovascularization, p18 interaction with the Rab4-recycling endosome is actively involved in new vessel formation [43]. In contrast to these studies, Rab11A and its effector, Rab11A FIP2, were found to be required for VE-cadherin recycling [44]. Depletion of Rab11A induced prolonged vascular leakage during polymicrobial septicemia via cecal ligation and puncture (CLP) by disrupting VE-cadherin recycling [44]. Thus, Rabs traffic and/or recycle proteins involved in endothelial barrier maintenance and control endothelial permeability. 

### 2.3. Novel Rab GTPases of Relevance to Endothelial Function

A unique function for a Rab GTPase found in endothelial cells is performed by Rab28. In arteries, from hypertensive arteries and endothelial cells after in vitro cyclic-strain-induced mechanical conditioning, an upregulation of Rab28 was observed [45]. This endothelial Rab28 colocalized with the p65 subunit of the transcription factor, NF-κB, and mediated its transport into the nucleus, where NF-κB was then released to act as a transcription factor for genes involved in endothelial proliferation [45] (Figure 1). 

A more recent discovery is that of large Rab GTPases [46]. While most Rab GTPases have a molecular weight between 20 and 35 kDa, these atypical or noncanonical Rabs have molecular weights between 70 and 150 kDa [46,47]. Three such isoforms have been identified, Rab44, Rab45, and Rab46 [47]. Rab46 was identified in T cells and endothelial cells [47]. The discovery of Rab46 was somewhat surprising given that its gene, CRACR2A (Ca^2+^-release-activated Ca^2+^ channel regulator 2A), was already shown to encode a 395-amino-acid protein which functions as a regulator for store-operated Ca^2+^ entry in T cells [48]. This isoform is now referred to as CRACR2A-S (or CRACR2A-c). Subsequently, it was confirmed that CRACR2A encodes a longer variant (731 aa) of the protein that contains a Rab domain in endothelial cells [49]. This variant is also known as CRACR2A-L (or CRACR2A-a). Due to the presence of a Rab domain, this variant was renamed Rab46 [50]. In endothelial cells, Rab46 colocalizes to WPBs, where it functions as a ‘Ca^2+^-sensing GTPase’ [50]. Upon acute histamine stimulation, Rab46 shuttles WPBs carrying noninflammatory cargo back to the microtubule-organizing center [50]. However, with continued histamine stimulation and an increase in intracellular Ca^2+^, Rab46 then functions to disperse WPBs and their cargo, thus modulating the endothelial cell response to inflammation [50] (Figure 1). Endothelial cells possess several unique functions; hence, it is more than likely that there are many more novel Rab GTPases in these cells. A summary of Rab GTPases in endothelial cells is provided in Table 1.

## 3. Rab GTPases and Their Involvement in Vascular Smooth Muscle Physiology and Pathophysiology

### 3.1. Rab GTPases in Ion Channel Subunit Trafficking

Our research group has uncovered the role of several Rab GTPases that mediate vascular smooth muscle function. Depolarization or hyperpolarization of the vascular smooth muscle plasma membrane (surface), which leads to constriction or dilation, respectively, is facilitated by smooth muscle ion channel function. The current *(I)* generated by a surface resident ion channel population is a product of *I=N.P_O_.i*, where *Po* is the single-channel open probability, and *i* is the single-channel current. *N* is the number of surface-resident channels, which was hypothesized to be modulated by acute and prolonged cellular stimuli.

Our research was on the large-conductance Ca^2+^-activated potassium (BK_Ca_, BK, K_Ca_1.1) channel α and β1 subunits. BK channels are homotetrameric assemblies of pore-forming α subunits [51], with at least two known accessory subunits in vascular smooth muscle, beta1 (β1) and LRRC26 (also known as γ1) [52,53]. These β1 and γ1 subunits associate with the α subunit to increase apparent Ca^2+^ sensitivity and voltage sensitivity, respectively, to control arterial contractility [52,53]. We first recorded that in cerebral and mesenteric artery smooth muscle cells, almost all BKα protein is surface-localized, while most β1 subunits, under resting conditions, are present intracellularly [54,55]. This suggested that β1 subunits are likely only trafficked to the surface in response to a stimulus. Since the nitric oxide (NO) pathway is a well-known stimulant of BK channel activity, we tested the possibility that NO signaling induced acute β1 surface trafficking in vascular smooth muscle. By using techniques such as surface biotinylation, FRET and RNAi, we uncovered that NO stimulated protein kinase G (PKG) signaling to induce very rapid (within seconds) surface trafficking of β1 subunits [55]. Overexpressing either the constitutively active Rab11A mutant (Rab11AQ70L) or the dominant negative Rab11A mutant (Rab11AS25N) increased or decreased surface β1 expression, respectively, which in turn altered BK channel activity [55]. 

Resistance arteries respond to increased intravascular pressure by depolarizing the plasma membrane and opening voltage-dependent Ca^2+^ channels (Ca_V_1.2), leading to Ca^2+^ influx and vasoconstriction [13,56]. This also triggers a burst release of Ca^2+^ from the sarcoplasmic reticulum, referred to as a ‘Ca^2+^ spark’, which activates BK channels, leading to vasodilation and thereby limiting Ca_V_1.2 activity [13,56,57]. Interestingly, this process also increased β1 surface trafficking through a Rab11A-dependent mechanism [58]. After we ruled out the involvement of PKG, we uncovered that Rho-associated protein kinase (ROCK) was involved in Rab11A activation and β1 surface trafficking [58]. This was surprising since ROCK activation is usually associated with vasoconstriction and suggested that pathways that cause vasoconstriction could also activate negative feedback loops to limit the contractile response. Overall, the data in these studies showed that NO- and pressure-induced depolarization mobilized the same pool of Rab11A+ endosome-associated β1 subunits, albeit via distinct mechanisms (NO through PKG and depolarization through ROCK activation) to induce BK channel activation. 

We then furthered these studies to investigate how this pathway was affected by disease. First, we discovered that the vasoconstrictor, endothelin-1, stimulated protein kinase C (PKC) activation, which phosphorylated serine177 on Rab11A and prevented its activation either by PKG or ROCK [59]. Later, in cerebral arteries of spontaneous-stroke-prone hypertensive rats (SP-SHR), we found that both NO- and depolarization-induced Rab11A-mediated β1 surface trafficking was severely restricted due to an upregulation of PKC signaling [60]. This inhibitory phosphorylation of Rab11A was immediately erased when a PKC inhibitor was introduced [60]. These studies showed that vascular smooth muscle Rab11A function was differentially regulated by protein kinases arising from disparate signaling networks.

In arteries, BKα subunits were not associated with Rab11A or Rab11B but with Rab4+ endosomes and, unlike β1 subunits, were constitutively trafficked to the cell surface [54]. The extended presence of angiotensin II, a vasoconstrictor, activated PKC-dependent internalization of BKα, which was then routed for degradation and not recycled back to the surface [54]. Together, these findings suggest that in diseases such as hypertension, where there is an increase in circulating vasoconstrictors such as endothelin-1 or angiotensin II, activation of PKC in vascular smooth muscle not only inhibits β1 recycling but also internalizes and degrades BK channel protein itself, thereby limiting smooth muscle relaxation. 

Interestingly, in HL-1-immortalized mouse atrial myocytes, the voltage-activated K^+^ channel (Kv1.5) was internalized and recycled back to the surface in a process involving Rab11 and Rab4 [61]. Ca_V_1.2 α subunits, similar to BKα, were also primarily cell-surface-localized in arterial smooth muscle [62]. In cerebral arteries, Ca_V_1.2 surface trafficking was regulated by Rab25 [62]. Rab25 is a member of the same family of Rabs, which includes Rab11A and Rab11B [1], but neither Rab11A nor Rab4 protein knockdown interfered with Ca_V_1.2 surface trafficking [62]. Knockdown of Rab25 triggered both lysosomal and proteasomal degradation of the Ca_V_1.2α subunits [62]. Thus, Rab GTPases influence smooth muscle reactivity by modulating the density of surface resident ion channels.

### 3.2. Rab GTPases’ Involvement in Other Smooth Muscle Functions

Apart from those above-mentioned, few other Rab GTPases have been described in vascular smooth muscle. Smooth muscle cell (SMC)-derived extracellular vesicles (EVs) have been implicated in arterial calcification [63]. While previously believed to be only engaged in intracellular protein sorting, sortilin was identified as the critical mediator for sorting the calcification protein, tissue-nonspecific alkaline phosphatase (TNAP), into Rab11A+ endosomes for secretion from SMCs [64]. In resting human aortic SMCs (HASMCs), Jagged1, one of the cell surface receptors for the Notch signaling pathway, colocalized with Rab4A+ endosomes, which suggested that this receptor is recycled in these cells [65]. Microarray analysis of human tissue revealed that Rab1A is enriched in smooth muscle, while Rab27B was found in uterine muscle [66]. Rab1 mediates intracellular protein transport from the endoplasmic reticulum to the Golgi apparatus [66] (Figure 1). Overexpression of wild-type (WT) Rab1 the increased surface expression of the angiotensin II (Ang II) type 1 receptor (AT1R) in rat pulmonary artery smooth muscle cells (RPASMs) [67]. In vitro hypoxia triggered Rab1 expression and appeared to regulate phenotypic switching of RPASMs [67]. Similarly, Rab6A was also induced by hypoxic stress and promoted vascular smooth muscle cell (VSMC) phenotypic switching [68]. All three Rab5 isoforms (A-C) are involved in the endosomal pathway and are ubiquitously expressed. In an intimal hyperplasia rat model, Rab5A was upregulated in thoracic aorta SMCs, while knockdown of Rab5A inhibited smooth muscle proliferation and migration [69]. Similarly, upregulation of Rab7 in SMCs of patients with acute aortic dissection (AAD) promoted VSMC proliferation and invasion [70]. In HASMCs, 17β-estradiol activated sirtuin-1 to induce Rab9-dependent autophagy of mitochondria and promote mitochondrial quality control, which delayed cellular senescence [71]. A summary of Rab GTPases in VSM is shown in Table 2. Although very limited, research into Rab GTPase function in vascular smooth muscle has revealed that these proteins are essential modulators of SMC function in physiology and pathophysiology.

## 4. Conclusions

Rab GTPases are ubiquitously expressed proteins that control various aspects of cellular function, from cell survival to secretion. These proteins, as summarized in Figure 1 and Table 1, Table 2 and Table 3, have vital roles to play in vascular endothelial and smooth muscle cells. However, their roles are often very fluid and, in many cases, multiple Rabs seemingly perform the same function. The lack of precise pharmacological tools compounded by the lethality of gene knockouts has limited the scope of investigations and understanding of the roles of individual Rabs. While at least endothelial cell Rab GTPase function has been explored in some detail, much remains to be learned about Rab function in vascular smooth muscle. Thus, studies into Rab GTPases will continue to be an exciting field for vascular biologists.

## Figures and Tables

**Table 1:** Rab GTPases identified in endothelial cells and their function.

Rab GTPase	Function
Rab1	β2-adrenergic receptor trafficking angiotensin II type 1 receptor trafficking
Rab3 (A, B, D) Rab3D	WPBs-vWF exocytosis Inhibits tubulogenesis
Rab4	VEGFR2 recycling VEGFR1 facilitates Rab4-integrin recycling VE-cadherin recycling
Rab5	KCNN3, KCNN4 endocytosis VEGFR2 recycling β1 integrin recycling (Rab5C)
Rab7	VEGFR2 trafficking to the lysosome Upregulation in high-fat diet
Rab8A	endothelial tubulogenesis
Rab11A	endothelial tubulogenesis VE-cadherin recycling
Rab13	Autophagy
Rab15	WPBs-vWF exocytosis
Rab27 Rab27B/7A	WPBs-vWF exocytosis miR-143 exosome secretion
Rab28	NF-κB nuclear transport
Rab33	Identified in WPBs but unknown function
Rab35	WPBs-vWF exocytosis KCNN3 recycling
Rab37	Identified in WPBs but unknown function
Rab46	Novel large Rab GTPase involved in WPB reorganization

**Table 2:** Rab GTPases in vascular smooth muscle.

Rab GTPase	Function
Rab1	angiotensin II type 1 receptor trafficking
Rab4	BK (K_Ca_1.1) channel α subunit surface trafficking, Kv1.5 channel trafficking, Jagged1 recycling
Rab5A	SM proliferation
Rab6A	Induction in hypoxic stress: Possible role in SM phenotypic switching
Rab9	Sirtuin-induced mitochondrial autophagy
Rab11	11A-BK (K_Ca_1.1) channel β1 subunit surface trafficking, tissue-nonspecific alkaline phosphatase exocytosis, Kv1.5 channel trafficking
Rab25	Ca_V_1.2 channel α subunit trafficking

**Table 3:** Rab GTPases function uncovered from in vivo studies.

Rab GTPase	Disease
Rab5A	Upregulated in aortic SMCs from intimal hyperplasia rat model
Rab7	Upregulated in patients with acute aortic dissection
Rab11A	Depletion causes prolonged vascular leakage by disruption of VE-cadherin recycling in CLP model
Rab11A	Decreased expression and PKC inhibition in arteries of hypertensive mice
Rab28	NF-κB nuclear transport, upregulation observed in hypertensive arteries

## Data Availability

Not applicable.
